# Advances in Biliary Tract Disorders: Novel Biomarkers, Pharmacotherapies, Endoscopic Techniques, and Surgical Management

**DOI:** 10.1155/2016/1583486

**Published:** 2016-12-18

**Authors:** Mohamad H. Imam, Sooraj Tejaswi, Mohammed Nabil Quraishi, James H. Tabibian

**Affiliations:** ^1^Division of Gastroenterology and Hepatology, University of Tennessee Health and Science Center, Memphis, TN, USA; ^2^Division of Gastroenterology and Hepatology, University of California, Davis, Sacramento, CA, USA; ^3^Department of Gastroenterology, University Hospital Birmingham, Birmingham, UK

Biliary tract disorders encompass a wide range of benign and malignant disease processes. In this special issue, we aimed to highlight advances in novel biomarkers, pharmacotherapies, endoscopic techniques, and surgical management of a variety of biliary tract disorders. Such advances continue to lead to improved diagnostic accuracy, better therapies, and subsequently superior patient outcomes. Manuscripts selected for publication in this issue come from around the world and address clinically relevant topics and advances in biliary tract disorders, as briefly summarized below.

Endoscopic retrograde cholangiopancreatography (ERCP) is an established procedure with numerous applications in biliary tract disorders. However, it may be associated with several periprocedural complications, including acute pancreatitis and cholangitis. The presence of periampullary diverticula (PAD) may influence the technical success and safety of ERCP and hence affect patient outcomes. Z. Sun et al. evaluate the difference in clinical outcomes between 161 patients with PAD as compared to matched controls.

Technical modifications and ancillary techniques to ERCP are areas of ongoing research. A strategy that involves placement of a biliary stent to avoid sphincterotomy in patients undergoing ERCP for common bile duct stones is described by T. Ueda et al.; preserving papillary integrity via such a technique may be especially desirable in younger patients. The use of endoscopic ultrasound (EUS) guided biliary drainage as an alternative treatment for biliary obstruction in cases of failed ERCP is growingly implemented; J. Guo et al. describe the outcomes of their experience utilizing this approach for the management of biliary obstruction. T. A. P. Franzini et al. provide an overview of various cholangioscopy techniques and comment on recent advances in visualization of the biliary system and its application to biliary disease management.

Evolution of specific surgical techniques such as those employed in Roux-en-Y hepaticojejunostomy is reviewed from a technical standpoint by D. Moris et al. They summarize their 25-year experience with RYHJ for management of bile duct injury and different perioperative measures they implement to optimize patient outcomes.

Various malignancies have been associated with primary biliary diseases. Optimization of surveillance strategies and care for these patients is crucial. A review manuscript by V. Hrad et al. summarizes the cancer risks found in different primary biliary diseases, including primary sclerosing cholangitis, primary biliary cholangitis, and overlap syndrome. This is complemented by a review paper in which L. Z. C. T. Pu et al. discuss evidence for best practice in management of malignant biliary strictures. For unresectable intrahepatic cholangiocarcinoma, treatment options are limited and often disappointing.

R. Seidensticker et al. aim to assess the outcomes of patients with unresectable intrahepatic cholangiocarcinoma treated by a tailored therapeutic approach, combining systemic and advanced image-guided local or locoregional therapies.

Lastly, gallstone disease remains a major public health burden worldwide. A. Hayasaki et al. aim to identify significant independent variables influencing postoperative hospital stay and medical costs in patients with definite, suspected, or unmatched acute cholecystitis diagnosis according to the 2013 Tokyo Guidelines.

We hope the manuscripts in this special issue will highlight useful advances in the field and help lay groundwork for further studies addressing the etiopathogenesis, diagnosis, and management of biliary tract disorders.

*Mohamad H. Imam* *Mohamad H. Imam* *Sooraj Tejaswi* *Sooraj Tejaswi* *Mohammed Nabil Quraishi* *Mohammed Nabil Quraishi* *James H. Tabibian* *James H. Tabibian*

